# Tissue of origin prediction for cancer of unknown primary using a targeted methylation sequencing panel

**DOI:** 10.1186/s13148-024-01638-6

**Published:** 2024-02-09

**Authors:** Miaomiao Sun, Bo Xu, Chao Chen, Youjie Zhu, Xiaomo Li, Kuisheng Chen

**Affiliations:** 1 Department of Pathology, Henan Key Laboratory of Tumor Pathology, The First Affiliated Hospital of Zhengzhou University, Zhengzhou, China; 2 Research and Development Division, Oriomics Biotech Inc, Hangzhou, China

**Keywords:** Cancer of unknown primary, Methylation classifier, Tissue of origin, Machine learning, Random forest, Elastic net, Targeted methylation sequencing

## Abstract

**Rationale:**

Cancer of unknown primary (CUP) is a group of rare malignancies with poor prognosis and unidentifiable tissue-of-origin. Distinct DNA methylation patterns in different tissues and cancer types enable the identification of the tissue of origin in CUP patients, which could help risk assessment and guide site-directed therapy.

**Methods:**

Using genome-wide DNA methylation profile datasets from The Cancer Genome Atlas (TCGA) and machine learning methods, we developed a 200-CpG methylation feature classifier for CUP tissue of origin prediction (MFCUP). MFCUP was further validated with public-available methylation array data of 2977 specimens and targeted methylation sequencing of 78 Formalin‐fixed paraffin‐embedded (FFPE) samples from a single center.

**Results:**

MFCUP achieved an accuracy of 97.2% in a validation cohort (*n* = 5923) representing 25 cancer types. When applied to an Infinium 450 K array dataset (*n* = 1052) and an Infinium EPIC (850 K) array dataset (*n* = 1925), MFCUP achieved an overall accuracy of 93.4% and 84.8%, respectively. Based on MFCUP, we established a targeted bisulfite sequencing panel and validated it with FFPE sections from 78 patients of 20 cancer types. This methylation sequencing panel correctly identified tissue of origin in 88.5% (69/78) of samples. We also found that the methylation levels of specific CpGs can distinguish one cancer type from others, indicating their potential as biomarkers for cancer diagnosis and screening.

**Conclusion:**

Our methylation-based cancer classifier and targeted methylation sequencing panel can predict tissue of origin in diverse cancer types with high accuracy.

**Supplementary Information:**

The online version contains supplementary material available at 10.1186/s13148-024-01638-6.

## Introduction

Cancer of unknown primary (CUP), accounting for about 2% of all cancer diagnoses, is a heterogeneous group of metastatic malignancies without identifiable primary tumor sites. CUP can be categorized into favorable and unfavorable subsets [1, 2]. Through a standard diagnostic workup, 15–20% of patients with CUP can be assigned to a putative primary tumor site [3]. Patients in these subsets typically receive site-specific therapies and have favorable outcome. The favorable-CUP subsets encompass head and neck squamous cell carcinoma, breast, ovarian, prostate, kidney, and colorectal cancer [1]. The remaining patients with CUP (80–85%) fall into the unfavorable subset and will receive empiric chemotherapies [3]. The favorable-CUP and unfavorable-CUP have median overall survivals (OS) of 11.7 months and 3.9 months, respectively [2]. The 1-year survival rates in these two subsets were 45% and 11%, respectively [2].

The initial evaluation for CUP includes a thorough physical examination, basic blood tests, CT/MRI scans, endoscopies, and microsatellite instability (MSI)/mismatch repair deficiency (dMMR) testing [3]. The major CUP histologies include well to moderately differentiated adenocarcinomas (~ 50%), poorly or undifferentiated adenocarcinomas (~ 30%), squamous-cell carcinomas (~ 15%), and undifferentiated neoplasms (~ 5%) [3]. Although a routine histopathological workout can determine the most likely cell lineages of CUP, it cannot define the primary tumor site for most CUP cases [4]. The identification of tissue of origin in patients with unfavorable-CUP can reassign them to the favorable-CUP subsets and enable the application of site-specific therapies [3].

Epigenetic modifications, including DNA methylation, play an important role in the regulation of tissue-specific gene expression and cellular identity [5]. Distinct DNA methylation pattern in different tissue and cancer types, making it a promising tool for cancer classification. The TCGA project has generated genome-wide DNA methylation profiles of 10,814 tumor samples in 33 cancer types [6]. This extensive methylation dataset enables the development of cancer classifiers, which can be used for CUP diagnosis [7].

DNA methylation profiling has been used in the classification of sarcoma, central nervous system (CNS) and sinonasal tumors [8–10]. Methylation classifiers also showed promising results in tissue of origin prediction among patients with CUP or head and neck squamous cell cancers with unknown primary (HNSCC-CUPs) [11, 12]. The primary goal of this study is to develop an affordable and accessible targeted methylation next-generation sequencing panel for CUP diagnosis. Furthermore, we discovered candidate CpGs whose methylation status can distinguish one cancer type from others.

## Methods

### Feature selection and classifier development

Whole-genome Illumina Infinium HumanMethylation450 (450 K) BeadChip array data across 22 cancer types and adjacent normal tissues were obtained from The Cancer Genome Atlas (TCGA) NCI GDC Data Portal (https://portal.gdc.cancer.gov) (Additional file 1: Table S1). Since the TCGA ovarian cancer methylation dataset was based on the low-coverage Infinium HumanMethylation27 (27 K) array, we replaced it with an ovarian cancer 450 K array methylation dataset (GSE102119) [13].

**Fig. 1:**
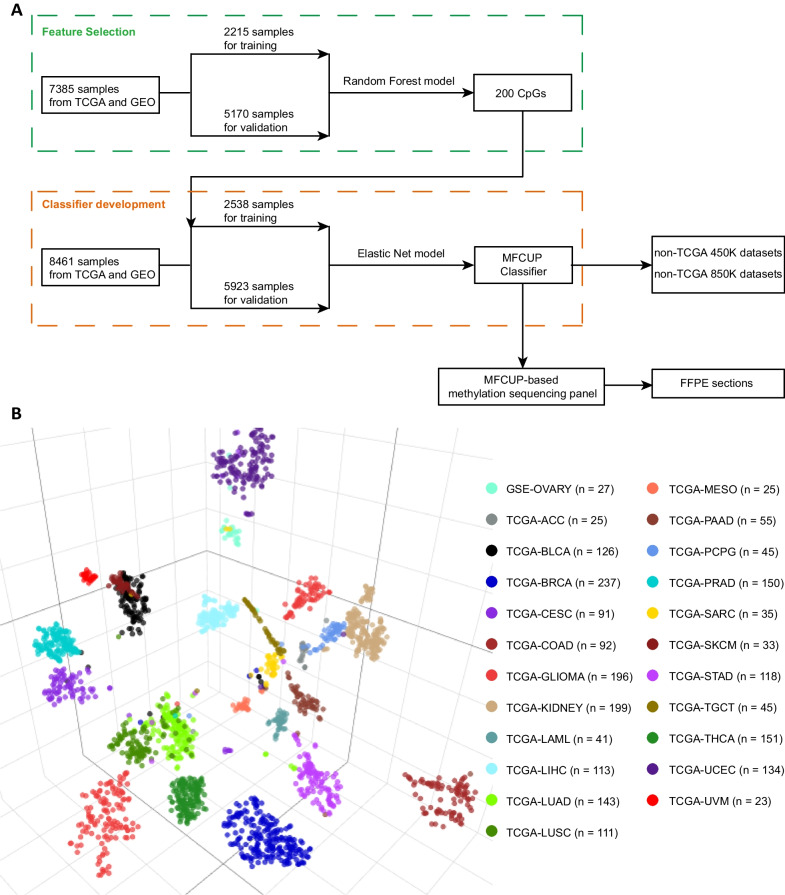
**A** Workflow for feature selection and classifier development. **B** *t*-distributed stochastic neighbor embedding (*t*-SNE) using methylation profiles of the 200 CpGs across the training cohort (*n* = 2215). *ACC* Adrenocortical carcinoma, *BLCA* Bladder urothelial carcinoma, *BRCA* Breast invasive carcinoma, *CESC* Cervical squamous cell carcinoma and endocervical adenocarcinoma, *COAD* Colon adenocarcinoma, *LAML* Acute myeloid leukemia, *LIHC* Liver hepatocellular carcinoma, *LUAD* Lung adenocarcinoma, *LUSC* Lung squamous cell carcinoma, *MESO* Mesothelioma, *PAAD* Pancreatic adenocarcinoma, *PCPG* Pheochromocytoma and paraganglioma, *PRAD* Prostate adenocarcinoma, *SARC* Sarcoma, *SKCM* Skin cutaneous melanoma, *STAD* Stomach adenocarcinoma, *TGCT* Testicular germ cell tumors, *THCA* Thyroid carcinoma, *UCEC* Uterine corpus endometrial carcinoma, *UVM* Uveal melanoma

For feature selection, we employed the Random Forest (RF) algorithm, which was used in the EPICUP CUP classifier and the DKFZ CNS tumor classifier [8, 12]. The combined methylation datasets of 23 cancer types were randomly split into a training set (30%) and a validation set (70%) (Fig. 1A, Additional file 1: Table S1). For every CpG site, an analysis of variance with one-way ANOVA was performed to compare methylation level (*β* values) among different cancer types. A Tukey’s honest significant difference post hoc test was applied to features with significant difference. CpGs that were differentially methylated in at least one cancer type were selected (Δ*β* > 0.2, *p* < 0.01). A RF classifying algorithm was then trained in two consecutive steps: (1) the selected CpGs were employed to build a prediction model using the RF machine learning method (R package randomForest version 4.7–1.1), and the variable importance of each CpG site was calculated by the mean decrease in accuracy; (2) CpGs with reduced out-of-bag (OOB) error were added in order of descending variable importance. We used default values of the RF parameters: ntree = 500, node size = 1, mtry = sqrt (*p*), where p is the number of features. After five runs of the two-step procedure, a total of 744 CpGs were obtained by the union of 200 CpGs with highest variable importance from each run. Next, we evaluated the tissue of origin prediction performance of the top 50, 100, 150, 200, 250, and 300 features on the validation set. We found that the top 50 features had the lowest accuracy (~ 96%), while others had similar results (~ 98%). In consideration of methylation signal loss during capture probes synthesis and targeted bisulfite sequencing, we chose 200 as the number of features for classifier development and targeted methylation sequencing panel design. We retrained the RF model with the 744 CpGs and sorted them with variable importance. The top 200 CpGs with highest variable importance were selected as the final methylation feature.

For classifier development, we applied 450 K methylation array datasets from 32 cancer types (31 from TCGA) (Additional file 1: Table S2). Based on the similarity of DNA methylomes and/or tissue of origins, we made the following adjustments: uterine carcinosarcoma (UCS) and uterine corpus endometrial carcinoma (UCEC) were grouped as the uterine cancer (UC) cohort (*n* = 368); colon and rectum adenocarcinoma (COAD/READ) were grouped as the colorectal cancer (CRC) cohort (*n* = 283); acute myeloid leukemia (LAML) and diffuse large B-cell lymphoma (DLBC) were grouped as the hematolymphoid malignancies (HLM) cohort (*n* = 134); esophageal and stomach adenocarcinoma (EAC/STAD) were grouped as the upper gastrointestinal tract adenocarcinoma cohort (Upper GI, *n* = 436); two squamous cell carcinoma datasets (ESCC/HNSC) were combined as the HN/ESCC cohort (*n* = 436). The expanded 25-cancer type datasets was randomly split into a training set (30%) and a validation set (70%) (Additional file 1: Table S2). Using the training set, we trained three different classifiers based on an RF, a Lasso, and an elastic net (EN) model. Because EN outperformed the other two on the validation set regarding prediction accuracy, sensitivity, and specificity, it was chosen as the final machine learning algorithm for classifier development.

### EN-based classifier validation with non-TCGA methylation array datasets

To further evaluate the performance of the EN-based methylation classifier, we employed non-TCGA 450K array data of 1,052 samples representing nine human cancers types (Additional file 1: Table S3), and Infinium EPIC (850K) array data of 1,925 specimens from 15 cancer types (Additional file 1: Table S4). For every tumor sample, the classifier generated a probability for each cancer types, and the tumor type with the highest probability was determined as the final prediction. Confusion matrixes were generated for all validation cohorts.

### Targeted bisulfite sequencing library preparation and sequencing

FFPE tumor tissue samples of 78 patients consisting 20 cancer types were retrospectively collected from The First Affiliated Hospital of Zhengzhou University. DNA was extracted from FFPE tumor tissues using TIANamp Genomic DNA Kit DP340 (Tiangen, Beijing, China). DNA extracts were sheared into 200–300-bp fragments using the Picoruptor (Diagenode, Liege, Belgium). Damaged bases of fragmented DNA were repaired with the NEBNext FFPE DNA Repair Mix Kit (NEB, Ipswich, MA, USA). The extracted DNA from FFPE sections was bisulfite-converted and purified using the EZ DNA Methylation-Gold Kit (Zymo Research, Orange, CA, USA). The bisulfite-converted DNA libraries were subsequently generated with an in-house protocol. An amount of 80 ng input DNA was found to be sufficient for the preparation of targeted bisulfite sequencing libraries. And we used it for all DNA sequencing samples.

Capture probes targeting the 200 selected CpGs were individually synthesized and 5’-biotinylated by Integrated DNA Technologies (IDT, Coralville, IA, USA). Hybridization capture-based target enrichment was carried out using NadPrep Hybrid Capture Reagents Kit (Nanodigmbio, Nanjing, China). Target capture libraries were sequenced on an Illumina NovaSeq 6000 sequencing platform.

### Methylation calling

The adapters, low-quality ends, and any sequencing reads less than 50 bp were removed by trim_galore (version 0.6.2). The reads were then mapped to the in-silico CT converted human RefSeq genome hg19 using Bismark (version 0.20.0). Duplicates were removed by the deduplicate_bismark module in Bismark. The methylation ratio for each CpG was calculated by the bismark_methylation_extractor script in Bismark.

## Results

### Methylation feature selection

Genome-wide Infinium 450 K DNA methylation array data of 7,385 tumor samples of known origin were obtained from TCGA (22 cancer types, *n* = 7294) and GSE102119 (ovarian cancer, *n* = 91) [6, 13]. Tumor samples were randomly assigned to the training (30%) and validation (70%) set. As described in methods, we chose the RF algorithm for feature selection and 200 as the number of features. The top 200 CpGs with highest variable importance were selected as the final feature for classifier development and targeted methylation sequencing panel design (Fig. 1A). A *t*-distributed stochastic neighbor embedding (*t*-SNE) dimensionality reduction plot showed the partition of different methylation classes representing 23 cancer types (Fig. 1B).

**Fig. 2:**
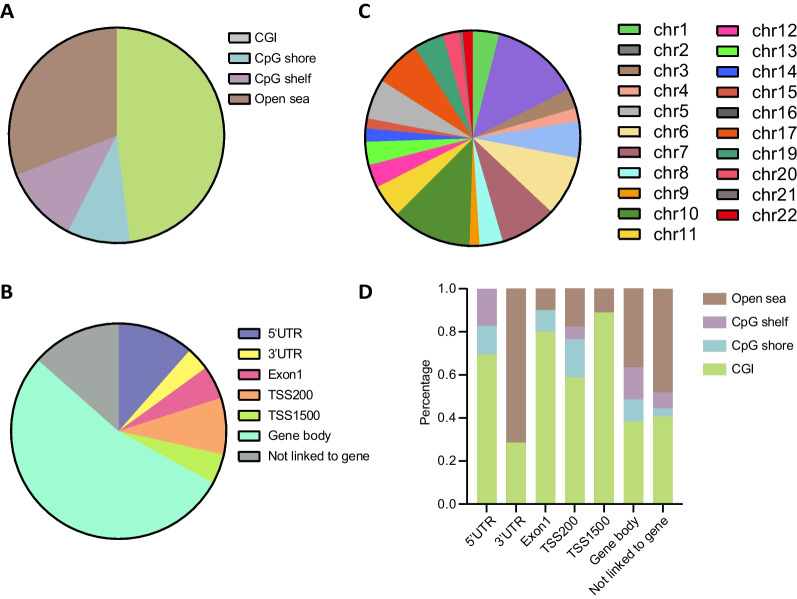
Distribution of 200 selected CpGs according to **A** CpG content and neighborhood context; **B** functional genomic regions(TSS200, TSS1500, 5’UTRs, first exons, gene bodies, and 3’UTRs); **C** chromosome location; **D** CpG content and functional genomic regions

Analysis of the 200 targeted CpG sites revealed that 48% are located in CpG islands, 21% in CpG shore/shelf regions, and 31% are in other regions of the genome without any enrichment of CpG content (open sea) (Fig. 2A). Upon inspection, these 200 CpGs are enriched in gene body region, evenly distributed across promoter, 5’UTR and intergenic region, and underrepresented for the 3’UTR (Fig. 2B). The 200 CpGs are distributed among all autosomes except for chr 18 (Fig. 2C). As shown in Fig. 2D, promoter probes are most enriched in CGIs, and less enriched in CpG shelves and open sea.

**Fig. 3:**
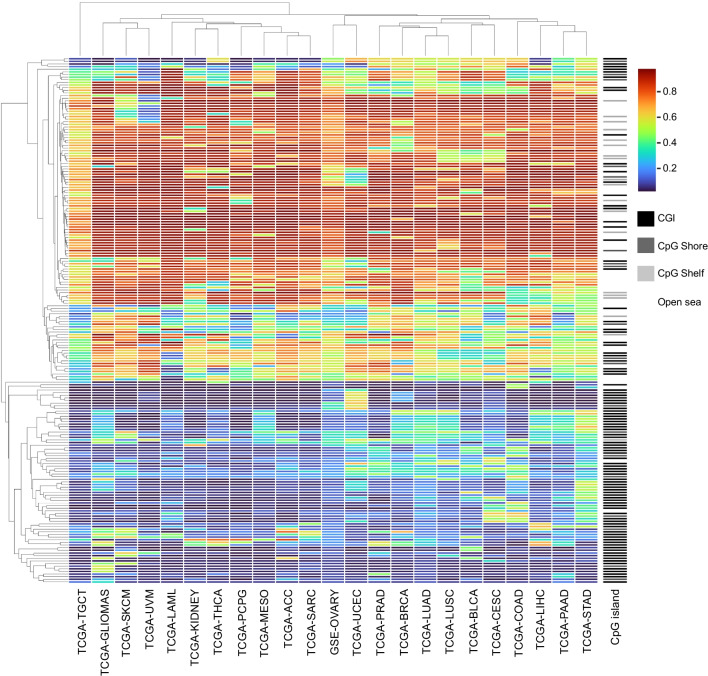
Heatmap of average methylation *β* values of the 200 CpGs resulting from 23 cancer types. CpG probes in rows and cancer-types in columns are hierarchically clustered. *ACC* Adrenocortical carcinoma, *BLCA* Bladder urothelial carcinoma, *BRCA* Breast invasive carcinoma, *CESC* Cervical squamous cell carcinoma and endocervical adenocarcinoma, *COAD* Colon adenocarcinoma, *LAML* Acute myeloid leukemia, *LIHC* Liver hepatocellular carcinoma, *LUAD* Lung adenocarcinoma, *LUSC* Lung squamous cell carcinoma, *MESO* Mesothelioma, *PAAD* Pancreatic adenocarcinoma, *PCPG* Pheochromocytoma and paraganglioma, *PRAD* Prostate adenocarcinoma, *SARC* Sarcoma, *SKCM* Skin cutaneous melanoma, *STAD* Stomach adenocarcinoma, *TGCT* Testicular germ cell tumors, *THCA* Thyroid carcinoma, *UCEC* Uterine corpus endometrial carcinoma, *UVM* Uveal melanoma

Clustering analysis of the training DNA methylation dataset revealed that hypomethylated CpGs are enriched in CpG islands, and hypermethylated CpGs are enriched in CpG shelfs/shores and open sea, respectively. Tumors originating from the same tissue or organ tended to cluster (Fig. 3). These included melanoma of the skin and eye (SKCM/UVM), and two lung cancers (LUAD and LUSC). The gastrointestinal carcinomas (COAD, LIHC, PAAD, and STAD) grouped together. Two adrenal gland tumors (PCPG and ACC) also grouped closely with the combined kidney tumors (KIDNEY). Two squamous cell carcinomas (LUSC and CESC) associated closely (Fig. 3).

**Fig. 4:**
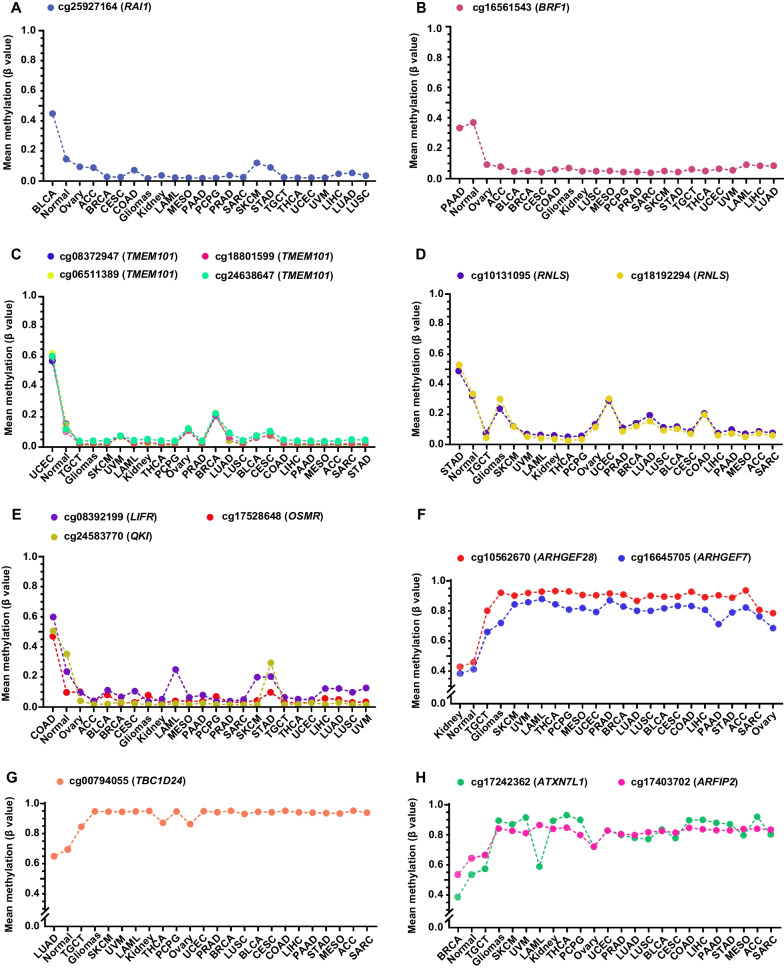
Examples of highly informative hyper- (**A**–**E**) and hypo- (**F**–**H**) methylation markers for specific cancer types. Mean methylation (*β* value) across 23 cancer types and the corresponding normal tissue were shown. *ACC* Adrenocortical carcinoma, *BLCA* Bladder urothelial carcinoma, *BRCA* Breast invasive carcinoma, *CESC* Cervical squamous cell carcinoma and endocervical adenocarcinoma, *COAD* Colon adenocarcinoma, *LAML* Acute myeloid leukemia, *LIHC* Liver hepatocellular carcinoma, *LUAD* Lung adenocarcinoma, *LUSC* Lung squamous cell carcinoma, *MESO* Mesothelioma, *PAAD* Pancreatic adenocarcinoma, *PCPG* Pheochromocytoma and paraganglioma, *PRAD* Prostate adenocarcinoma, *SARC* Sarcoma, *SKCM* Skin cutaneous melanoma, *STAD* Stomach adenocarcinoma, *TGCT* Testicular germ cell tumors, *THCA* Thyroid carcinoma, *UCEC* Uterine corpus endometrial carcinoma, *UVM* Uveal melanoma

The clustering heatmap of the 200 selected CpGs revealed that the methylation level of some CpGs can distinguish one cancer type from others, indicating their potential as biomarkers. For instance, cg25927164 (*RAI1*) and cg16561543 (*BRF1*) were hypermethylated in muscle-invasive bladder cancer and pancreatic ductal adenocarcinoma, respectively (Fig. 4A, B). CpGs located in the same CGI of *TMEM101* (*n* = 4) and *RNLS* (*n* = 2) were hypermethylated in uterine corpus endometrial carcinoma and stomach adenocarcinoma, respectively ( Fig. 4 C, D). The 200 CpGs also included three known colon cancer-specific biomarkers (*LIFR*, *OSMR*, *QKI*) (Fig. 4E) [14–16]. Interestingly, CpGs in two genes encoding guannine nucleotide exchanging factors for Rho GTPase (*ARHGEF28* and *ARHGEF7*) were hypomethylated in kidney cancer (Fig. 4F) [17]. cg00794055 in *TBC1D24,* which encodes a putative Rab35-GTPase activating protein (Rab35-GAP), was hypomethylated in lung adenocarcinoma (LUAD) (Fig. 4G) [18]. cg17242362 (*ATXN7L1*) and cg17403702 (*ARFIP2*) were hypomethylated in breast cancer alone (Fig. 4H). Consistent with previous reports, *HOXA9* (cg16104915) was hypermethylated in cutaneous melanoma, lung, bladder, breast, and ovarian cancer [19–23] (Additional file 1: Figure S1).

### Classifier development with the elastic net algorithm

For methylation classifier development, we employed 31 out of 33 available TCGA methylation datasets. The original TCGA esophageal carcinoma (ESCA) study recommended treating esophageal adenocarcinoma (EAC) and squamous cell carcinoma (ESCC) as two entities [24]. Consistently, the TCGA pan-cancer cell-of-origin study revealed that EAC clustered tightly with stomach adenocarcinoma (STAD), while head and neck squamous cell carcinoma (HNSC) and ESCC formed a Pan-Squamous cluster [6]. Based on the latter work, we combined two colorectal cancers (COAD and READ), two uterine cancers (UCS and UCEC), two upper gastrointestinal tract cancers (EAC and STAD), two squamous cell carcinoma datasets (ESCC and HNSC), and two hematolymphoid maligancies (LAML and DLBC) in downstream analysis.

**Fig. 5:**
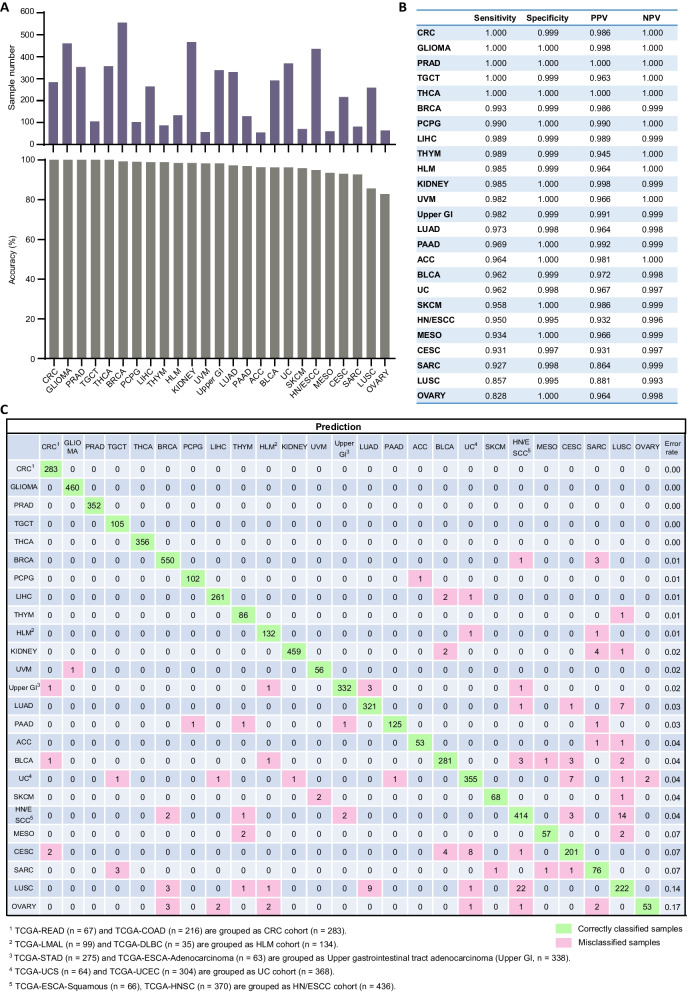
Cancer type classification accuracy of the expanded TCGA/GSE validation cohort (*n* = 5923). **A** Sample number and prediction accuracy (%) of each cancer type. **B** Sensitivity, specificity, PPV, and NPV for each of the 25 cancer types. **C** Confusion matrix (in percent) of the expanded TCGA/GSE validation cohort of cancer type prediction using 200 selected probes. The percentages of correctly predicted samples are highlighted in green; misclassification events are highlighted in pink. True histology/predicted histology is respectively listed in rows/columns. *ACC* Adrenocortical carcinoma, *BLCA* Bladder urothelial carcinoma, *BRCA* Breast invasive carcinoma, *CESC* Cervical squamous cell carcinoma and endocervical adenocarcinoma; *CRC* Colorectal cancer, *HLM* Hematolymphoid malignancies, *HN/ESCC* Head and neck squamous carcinoma and esophageal squamous cell carcinoma, *LIHC* Liver hepatocellular carcinoma, *LUAD* Lung adenocarcinoma, *LUSC* Lung squamous cell carcinoma, *MESO* Mesothelioma, *PAAD* Pancreatic adenocarcinoma, *PCPG* Pheochromocytoma and paraganglioma, *PRAD* Prostate adenocarcinoma, *SARC* Sarcoma, *SKCM* Skin cutaneous melanoma, *TGCT* Testicular germ cell tumors, *THCA* Thyroid carcinoma, *THYM* Thymoma, *UC* Uterine cancer, *Upper GI* Upper gastrointestinal adenocarcinoma, *UVM* Uveal melanoma

The expanded TCGA/GSE dataset was randomly split into the training set (30%) and validation set (70%) (Additional file 1: Table S2). Based on the 200-CpG probe set, we developed three different classifiers with an RF, a Lasso, and an elastic net (EN) model on the training set. As EN outperformed the other two models on the validation set, it was selected as the final algorithm for classifier development. The EN-based classifier MFCUP predicted the tissue of origin with an overall accuracy of 97.2% in the validation set (Fig. 5A). The sensitivity, specificity, positive and negative predictive values (PPVs and NPVs) for each of the 25 cancer types were shown in Fig. 5B. MFCUP achieved a prediction accuracy of 100% for CRC, GLIOMA, PRAD, TGCT and THCA (Fig. 5A, B, C). Methylation classes represent different cancer types in the validation set also separated well in the *t*-distributed stochastic neighbor embedding (*t*-SNE) dimensionality reduction diagram (Additional file 1: Figure S2).

### Classifier evaluation with non-TCGA methylation array datasets

**Fig. 6:**
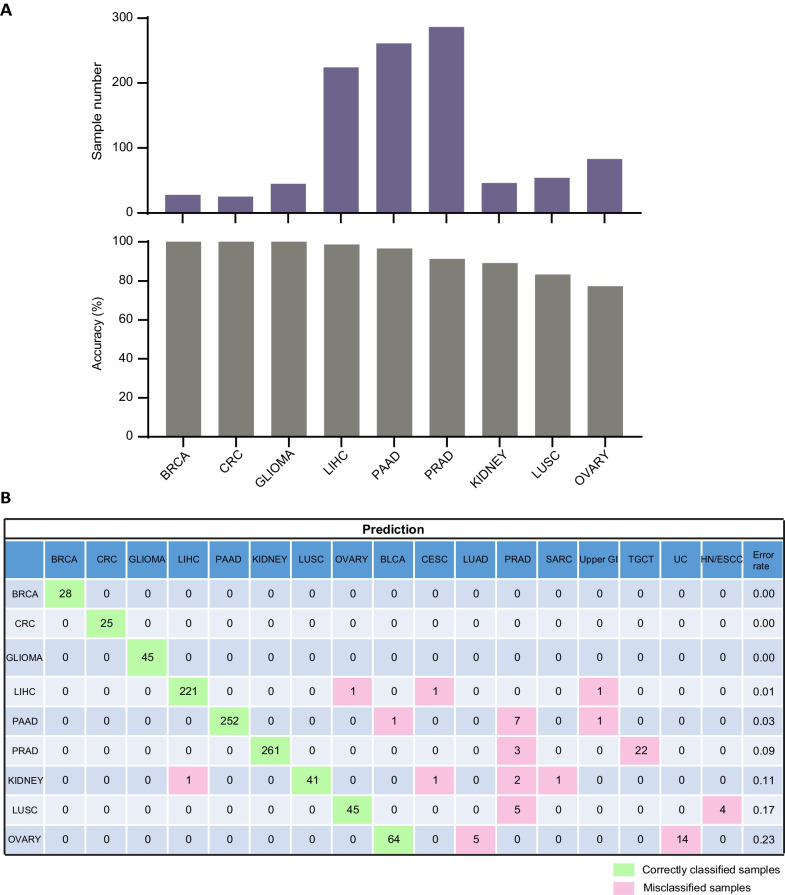
Cancer type classification accuracy of the Infinium 450 K array testing datasets. **A** Sample number and prediction accuracy (%) of nine cancer types. **B** Confusion matrix (in percent) of the cancer type prediction using 200 selected probes for testing datasets generated by infinium 450 K methylation array. The percentages of correctly predicted samples are highlighted in green; misclassification events are highlighted in pink. True histology/predicted histology is respectively listed in rows/columns. *BLCA* Bladder urothelial carcinoma, *BRCA* Breast invasive carcinoma, *CESC* Cervical squamous cell carcinoma and endocervical adenocarcinoma; *CRC* Colorectal cancer, *HN/ESCC* Head and neck squamous carcinoma and esophageal squamous cell carcinoma, *LIHC* Liver hepatocellular carcinoma, *LUAD* Lung adenocarcinoma, *LUSC* Lung squamous cell carcinoma, *PAAD* Pancreatic adenocarcinoma, *PRAD* Prostate adenocarcinoma, *SARC* Sarcoma, *TGCT* Testicular germ cell tumors, *THYM* Thymoma, *UC* Uterine cancer, *Upper GI* Upper gastrointestinal adenocarcinoma

**Fig. 7:**
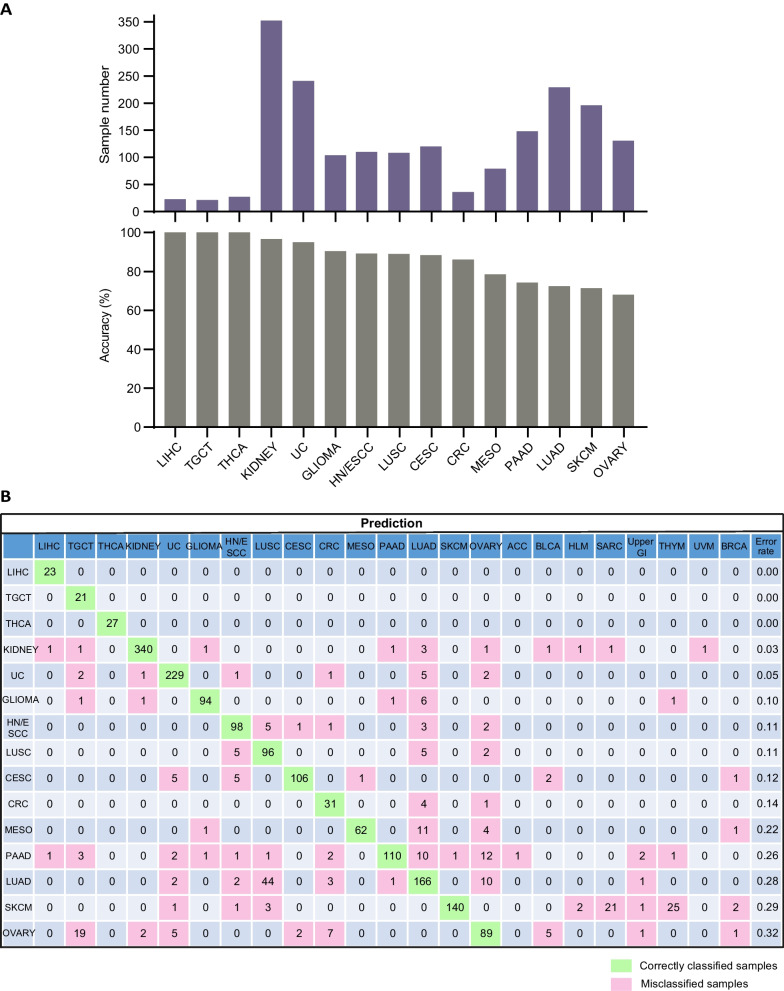
Cancer type classification accuracy of the Infinium 850 K array testing datasets. **A** Sample number and prediction accuracy (%) of 15 cancer types. **B** Confusion matrix (in percent) of the cancer type prediction using 200 selected probes for testing datasets generated by infinium 850 K methylation array. The percentages of correctly predicted samples are highlighted in green; misclassification events are highlighted in pink. True histology/predicted histology is respectively listed in rows/columns. *ACC* Adrenocortical carcinoma, *BLCA* Bladder urothelial carcinoma, *CESC* Cervical squamous cell carcinoma and endocervical adenocarcinoma, *CRC* Colorectal cancer, *HLM* Hematolymphoid malignancies, *HN/ESCC* Head and neck squamous carcinoma and esophageal squamous cell carcinoma, *LIHC* Liver hepatocellular carcinoma, *LUAD* Lung adenocarcinoma, *LUSC* Lung squamous cell carcinoma, *MESO* Mesothelioma, *PAAD* Pancreatic adenocarcinoma, *PCPG* Pheochromocytoma and paraganglioma, *PRAD* Prostate adenocarcinoma, *SARC* Sarcoma, *SKCM* Skin cutaneous melanoma, *TGCT* Testicular germ cell tumors, *THCA* Thyroid carcinoma, *UC* Uterine cancer, *Upper GI* Upper gastrointestinal adenocarcinoma, *UVM* Uveal melanoma

To explore the performance of MFCUP on non-TCGA methylation datasets, we validated it with Infinium 450 K array data of 1,052 tumor samples of nine cancer types obtained from the International Cancer Genome Consortium (ICGC) and GEO (Additional file 1: Table S3) (ICGC Data Portal https://dcc.icgc.org/) [25–30]. For this dataset, MFCUP achieved an overall accuracy of 93.4% (Fig. 6). Next, we evaluated its performance with Infinium EPIC/850 K array data of 1,925 tumor samples of 15 cancer types obtained from the Clinical Proteomic Tumor Analysis Consortium (CPTAC) and GEO (Additional file 1: Table S4) [31–40]. For the 850 K dataset, MFCUP achieved a classification accuracy of 84.8% (1632/1925) (Fig. 7).

### MFCUP-based methylation sequencing panel

**Fig. 8:**
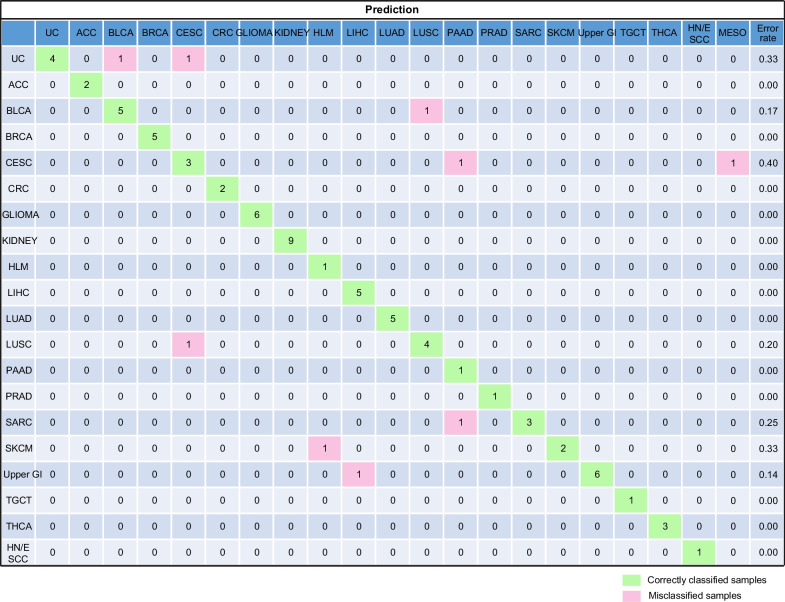
Confusion matrix (in percent) of the validation set of FFPE tumor tissues from 20 cancer types. Confusion matrix of the validation set (*n* = 78) of cancer type prediction using 200 selected probes. The percentages of correctly predicted samples are highlighted in green; misclassification events are highlighted in pink. True histology/predicted histology is respectively listed in rows/columns. *ACC* Adrenocortical carcinoma, *BLCA* Bladder urothelial carcinoma, *BRCA* Breast invasive carcinoma, *CESC* Cervical squamous cell carcinoma and endocervical adenocarcinoma; *CRC* Colorectal cancer, *HLM* Hematolymphoid malignancies, *HN/ESCC* Head and neck squamous carcinoma and esophageal squamous cell carcinoma, *LIHC* Liver hepatocellular carcinoma, *LUAD* Lung adenocarcinoma, *LUSC* Lung squamous cell carcinoma, *MESO* Mesothelioma, *PAAD* Pancreatic adenocarcinoma, *PRAD* Prostate adenocarcinoma, *SKCM* Skin cutaneous melanoma, *TGCT* Testicular germ cell tumors, *THCA* Thyroid carcinoma, *UC* Uterine cancer, *Upper GI* Upper gastrointestinal adenocarcinoma

The major obstacles for methylation-based CUP diagnosis included high cost and the lack of DNA methylation array facilities in most hospitals. To overcome these challenges, we developed a targeted methylation sequencing panel based on the 200 CpGs set of MFCUP. We evaluated the performance of this panel with 78 FFPE samples from 20 cancer types, in which it achieved a classification accuracy of 88.5% (69/78) (Fig. 8).

## Discussion

Recent studies have shown that DNA methylation profiling can be a valuable aid for accurate diagnosis of cancers of nervous tissue and muscular tissue [7]. For example, central nervous system (CNS) cancers are a heterogeneous group of tumors consisting around 100 entities, which makes accurate diagnosis of CNS tumor difficult. The German Cancer Research Center (DKFZ) developed a clinical-grade CNS tumor classifier, which assigned a distinctive methylation signature to nearly all CNS tumor types [8, 9]. This classifier was trained with 2,801 tumor samples comprising 91 methylation classes, and resulted in a diagnosis change in 12% of prospective CNS tumor cases [8]. Based on this work, DNA methylation‐based tumor classification is now included in the World Health Organization (WHO) classification of adult and pediatric CNS tumors [41, 42]. Sarcomas are a heterogeneous group of solid tumors of mesenchymal origin, which are difficult to diagnosis due to the lack of defining histopathological features in some subtypes. The DKFZ group also developed a methylation-based sarcoma classifier, which achieved a prediction accuracy of 75% in the validation sarcoma cohort (*n* = 428) [17]. In another validation study, the DKFZ sarcoma classifier was in accordance with the pathologic diagnosis in 88% of cases [43]. These results suggest that DNA methylation profile may provide greater diagnosis precision than standard protocols.

To extend methylation-based cancer classification beyond single tissue-of-origin, several groups developed multi-cancer classifiers with large methylation datasets and machine learning, but challenges remained [6, 44–46]. In a landmark study, Moran et al. established a DNA methylation-based CUP classifier, which can guide site-specific therapies for patients with CUP [12]. Using unsupervised clustering of methylation profile of 3,139 cancer-hypermethylated CpGs, Hoadley et al. divided 10,814 tumor samples from the TCGA dataset into 25 methylation groups [6]. Tang et al. [44] and Liu et al. [45] developed multi-cancer classifiers for tumor tissue/circulating-free DNA, respectively. However, these two classifiers target 5457/9223 CpGs, which were impractical in many clinical settings. Danilova et al. [46] developed a 305-CpG cancer classifier with a discovery set consisting five core cancer types. However, its prediction accuracy significantly decreased when applying to other cancer types. A cost-effective methylation sequencing panel, including dozens to hundreds excellent informative and discriminative CpG markers, is still lacking in the clinical practice of CUP diagnosis. Our aim was to develop an accessible and affordable DNA methylation-based CUP diagnosis assay independent of the high-throughput methylation array platform. Further studies are needed to evaluate the performance of our targeted methylation sequencing panel on metastases.

Human organs are highly complex and composed of multiple tissue and cell types. Genome-wide methylation profiling studies have revealed distinct methylation patterns in different human tissue and cell types [47, 48]. Tissue-specific DNA methylation patterns provide a useful tool for the characterization of tissue-of-origin [47, 49]. Similarly, cell type-specific DNA methylation profiles enable cell type deconvolution in tissue samples [47, 49]. Both tissue-specific and cancer-specific DNA methylation patterns appear to be maintained during cancer evolution [7]. A DNA methylation atlas based on deep whole-genome bisulfite sequencing of 39 normal human cell types demonstrated that almost all (97%) cell-type-specific differentially methylated regions (DMRs) are demethylated in one cell type but methylated in other cell types [47]. The authors suggested that this atlas can be used to identify the tissue of origin of cfDNA in plasma of cancer patients. 14% of these cell type specific DMRs are covered by the Infinium 450 K array [9]. Interestingly, three CpGs in our methylation classifier are located in cell-type-specific unmethylation regions described in the normal human cell methylome study [47], including breast luminal epithelium cell marker cg17403702 (*ARFIP2*), kidney epithelial cell marker cg10572670 (*ARHGEF28*), and lung alveolar epithelial cell marker cg00794055 (*TBC1D24*). Consistently, these CpGs are hypomethylated in one cancer type and the corresponding normal tissue, but hypermethylated in other cancer types. Moreover, our data showed that the lung alveolar epithelial cell DNA methylation biomarker cg00794055 (*TBC1D24*) are hypomethylated in LUAD and the corresponding normal control (LUAD), but hypermethylated in LUSC. Deconvolution of the TCGA LUAD/LUSC 450 K DNA methylation array datasets revealed that the relative proportion of lung alveolar epithelial cell in LUAD and normal adjacent tissue (LUAD and LUSC) are approximately 25% but less than 5% in LUSC [47]. This result explained why the methylation level of cg00794055 (*TBC1D24*) in LUSC was higher than LUAD.

Our work identified some validated cancer biomarkers. cg16104915, a CpG site located in the promoter CpG island of *HOXA9*, is a well-characterized biomarker in our 200-CpG set. It is methylated in 97% of NSCLC TCGA samples but not normal tissue [50]. *HOXA9* methylation is also a validated biomarker for cutaneous melanoma progression, with high methylation in metastases but low methylation in primary melanoma and nevi [20]. Our CpG set also included three known biomarker genes for colorectal cancer (*LIFR*, *OSMR*, *QKI*). The methylation levels of 10 CpGs in the *QKI* promoter were significantly higher in CRC than in normal tissues and other cancer types [16]. cg24583770 was adjacent to these 10 CpGs, and its hypermethylation status also distinguished colon cancer from normal tissues and other cancer types. Methylation of a segment of the *OSMR* promoter CGI (from -282 to -224) was found in 90% of colon cancer, 55% of normal-appearing mucosa adjacent to colon cancer, 33% of gastric cancer, and 20% of pancreatic cancer [51]. cg17528648 was in the 5’-UTR region of this *OSMR* CGI, and its hypermethylation distinguished colon cancer from adjacent normal mucosa and other cancer types. Hypermethylation of a CpG island located in the promoter of *HOXD8* (chr2:176,993,479–176,995,557) is a validated biomarker of biliary tract cancer [52]. (Additional file 2).

Through inspection of our 200-CpG set, we found some potential biomarkers for cancer type diagnosis. For instance, four CpGs within the same CpG island of *TMEM101*, a potential biomarker for reduced overall survival in breast cancer patients [53], were hypermethylated in UCEC but not in other cancer types. Similarly, cg25927164 (*RAI1*) was hypermethylated in BLCA only. Further studies are required to determine whether hypermethylation of *TMEM101* and *RAI1* could be used as biomarkers for the screen and diagnosis of endometrial and bladder cancer, respectively (Additional file 3).

## Conclusions

In summary, we developed a DNA methylation-based CUP classifier (MFCUP) with machine learning algorithms. To make DNA methylation-based diagnosis accessible and affordable, we established and validated a targeted methylation sequencing panel, which demonstrated high accuracy in identifying the primary sites for CUP. Lastly, our work revealed some CpGs with biomarker potential for cancer type classification.

### Supplementary Information

**Additional file 1.** Supplementary Table 1-4; Supplementary Figure 1-2.

**Additional file 2.** The β values of validation datasets.

**Additional file 3.** The corresponding code for this study.

## Data Availability

The code and data are available in the Additional files 2. The *β* values of validation datasets were provided. Other supporting data are available from the corresponding author upon reasonable request.

## Abbreviations

CUP: Cancer of unknown primary
CPTAC: Clinical Proteomic Tumor Analysis Consortium
CNS: Central nervous system
DMR: Differentially methylated regions
dMMR: Mismatch repair deficiency
EN: Elastic net
FFPE: Formalin‐fixed paraffin‐embedded
MFCUP: Methylation feature-based CUP classifier
MSI: Microsatellite instability
OS: Overall survival
RF: Random forest
TCGA: The Cancer Genome Atlas
*t*-SNE: *t*-Distributed stochastic neighbor embedding
ICGC: International Cancer Genome Consortium
WHO: World Health Organization
UTR: Untranslated region

## References

[1] Krämer A (2023). Cancer of unknown primary: ESMO clinical practice guideline for diagnosis, treatment and follow-up. Ann Oncol.

[2] Culine S (2002). Development and validation of a prognostic model to predict the length of survival in patients with carcinomas of an unknown primary site. J Clin Oncol.

[3] Rassy E, Pavlidis N (2020). Progress in refining the clinical management of cancer of unknown primary in the molecular era. Nat Rev Clin Oncol.

[4] Lee MS, Sanoff HK (2020). Cancer of unknown primary. BMJ.

[5] Dawson MA, Kouzarides T (2012). Cancer epigenetics: from mechanism to therapy. Cell.

[6] Hoadley KA (2018). Cell-of-origin patterns dominate the molecular classification of 10,000 tumors from 33 types of cancer. Cell.

[7] Papanicolau-Sengos A, Aldape K (2022). DNA methylation profiling: an emerging paradigm for cancer diagnosis. Annu Rev Pathol.

[8] Capper D (2018). DNA methylation-based classification of central nervous system tumours. Nature.

[9] Koelsche C (2021). Sarcoma classification by DNA methylation profiling. Nat Commun.

[10] Jurmeister P (2022). DNA methylation-based classification of sinonasal tumors. Nat Commun.

[11] Leitheiser M (2022). Machine learning models predict the primary sites of head and neck squamous cell carcinoma metastases based on DNA methylation. J Pathol.

[12] Moran S (2016). Epigenetic profiling to classify cancer of unknown primary: a multicentre, retrospective analysis. Lancet Oncol.

[13] Fang F (2018). Genomic and epigenomic signatures in ovarian cancer associated with resensitization to platinum drugs. Cancer Res.

[14] Li D (2022). Discovery and validation of tissue-specific DNA methylation as noninvasive diagnostic markers for colorectal cancer. Clin Epigenetics.

[15] Bedin C (2017). Diagnostic and prognostic role of cell-free DNA testing for colorectal cancer patients. Int J Cancer.

[16] Zhang L (2022). Promoter methylation of QKI as a potential specific biomarker for early detection of colorectal cancer. Front Genet.

[17] Cervantes-Villagrana RD, García-Jiménez I, Vázquez-Prado J (2023). Guanine nucleotide exchange factors for Rho GTPases (RhoGEFs) as oncogenic effectors and strategic therapeutic targets in metastatic cancer. Cell Signal.

[18] Yoon J (2018). TBC1d24-ephrinB2 interaction regulates contact inhibition of locomotion in neural crest cell migration. Nat Commun.

[19] Hulbert A (2017). Early detection of lung cancer using DNA promoter hypermethylation in plasma and sputum. Clin Cancer Res.

[20] Wouters J (2017). Comprehensive DNA methylation study identifies novel progression-related and prognostic markers for cutaneous melanoma. BMC Med.

[21] López JI (2017). A DNA hypermethylation profile reveals new potential biomarkers for the evaluation of prognosis in urothelial bladder cancer. APMIS.

[22] Singh A (2020). Detection of aberrant methylation of HOXA9 and HIC1 through multiplex methylight assay in serum DNA for the early detection of epithelial ovarian cancer. Int J Cancer.

[23] Park SM (2017). A long-range interactive DNA methylation marker panel for the promoters of HOXA9 and HOXA10 predicts survival in breast cancer patients. Clin Epigenetics.

[24] Integrated genomic characterization of oesophageal carcinoma (2017). Nature.

[25] Luo Y (2019). Regional methylome profiling reveals dynamic epigenetic heterogeneity and convergent hypomethylation of stem cell quiescence-associated genes in breast cancer following neoadjuvant chemotherapy. Cell Biosci.

[26] Qu X (2016). Integrated genomic analysis of colorectal cancer progression reveals activation of EGFR through demethylation of the EREG promoter. Oncogene.

[27] Zhang L (2014). Exome sequencing identifies somatic gain-of-function PPM1D mutations in brainstem gliomas. Nat Genet.

[28] Villanueva A (2015). DNA methylation-based prognosis and epidrivers in hepatocellular carcinoma. Hepatology.

[29] Wei JH (2015). A CpG-methylation-based assay to predict survival in clear cell renal cell carcinoma. Nat Commun.

[30] Teixeira VH (2019). Deciphering the genomic, epigenomic, and transcriptomic landscapes of pre-invasive lung cancer lesions. Nat Med.

[31] Liang WW (2023). Integrative multi-omic cancer profiling reveals DNA methylation patterns associated with therapeutic vulnerability and cell-of-origin. Cancer Cell.

[32] Cerapio JP (2021). Global DNA hypermethylation pattern and unique gene expression signature in liver cancer from patients with Indigenous American ancestry. Oncotarget.

[33] Fazal Z (2021). Hypermethylation and global remodelling of DNA methylation is associated with acquired cisplatin resistance in testicular germ cell tumours. Epigenetics.

[34] Park JL (2020). Comprehensive DNA methylation profiling identifies novel diagnostic biomarkers for thyroid cancer. Thyroid.

[35] Gagliardi A (2020). Analysis of Ugandan cervical carcinomas identifies human papillomavirus clade-specific epigenome and transcriptome landscapes. Nat Genet.

[36] Condelli V (2021). Novel epigenetic eight-gene signature predictive of poor prognosis and MSI-like phenotype in human metastatic colorectal carcinomas. Cancers.

[37] Bertero L (2021). DNA methylation profiling discriminates between malignant pleural mesothelioma and neoplastic or reactive histologic mimics. J Mol Diagn.

[38] Mitra S (2020). Analysis of DNA methylation patterns in the tumor immune microenvironment of metastatic melanoma. Mol Oncol.

[39] Gonzalez Bosquet J (2021). Creation and validation of models to predict response to primary treatment in serous ovarian cancer. Sci Rep.

[40] Vougiouklakis T (2022). Integrated analysis of ovarian juvenile granulosa cell tumors reveals distinct epigenetic signatures and recurrent TERT rearrangements. Clin Cancer Res.

[41] Louis DN (2021). The 2021 WHO classification of tumors of the central nervous system: a summary. Neuro Oncol.

[42] Pfister SM (2022). A summary of the inaugural WHO classification of pediatric tumors: transitioning from the optical into the molecular era. Cancer Discov.

[43] Lyskjaer I (2021). DNA methylation-based profiling of bone and soft tissue tumours: a validation study of the 'DKFZ Sarcoma Classifier'. J Pathol Clin Res.

[44] Tang W (2018). Tumor origin detection with tissue-specific miRNA and DNA methylation markers. Bioinformatics.

[45] Liu L (2018). Targeted methylation sequencing of plasma cell-free DNA for cancer detection and classification. Ann Oncol.

[46] Danilova L (2022). DNA-methylation for the detection and distinction of 19 human malignancies. Epigenetics.

[47] Loyfer N (2023). A DNA methylation atlas of normal human cell types. Nature.

[48] Schultz MD (2015). Human body epigenome maps reveal noncanonical DNA methylation variation. Nature.

[49] Zhu T (2022). A pan-tissue DNA methylation atlas enables in silico decomposition of human tissue methylomes at cell-type resolution. Nat Methods.

[50] Wrangle J (2014). Functional identification of cancer-specific methylation of CDO1, HOXA9, and TAC1 for the diagnosis of lung cancer. Clin Cancer Res.

[51] Deng G (2009). Unique methylation pattern of oncostatin m receptor gene in cancers of colorectum and other digestive organs. Clin Cancer Res.

[52] Loi E (2022). HOXD8 hypermethylation as a fully sensitive and specific biomarker for biliary tract cancer detectable in tissue and bile samples. Br J Cancer.

[53] Suman M (2021). Association of variably methylated tumour DNA regions with overall survival for invasive lobular breast cancer. Clin Epigenetics.

