# Supplementary material for: Tissue of origin prediction for cancer of unknown primary using a targeted methylation sequencing panel
Source: Clin Epigenetics. 2024 Feb 9;16:25. doi: 10.1186/s13148-024-01638-6 (PMC10854167; doi:10.1186/s13148-024-01638-6)

**Table S1 DNA methylation datasets used for feature selection**

| Project Name | Primary site | Pathological classification | Sample number |
| --- | --- | --- | --- |
| GEO-GSE102119 | Ovary | Ovarian Cancer | 91 |
| Kidney Renal Clear Cell Carcinoma (TCGA-KIRC)^1^ | Kidney | Adenomas and Adenocarcinomas | 324 |
| Kidney Renal Papillary Cell Carcinoma (TCGA-KIRP)^1^ | Kidney | Adenomas and Adenocarcinomas | 275 |
| Chromophobe Renal Cell Carcinoma (TCGA-KICH)^1^ | Kidney | Adenomas and Adenocarcinomas | 66 |
| Lung Squamous Cell Carcinoma (TCGA-LUSC) | Bronchus and lung | Squamous Cell Neoplasms | 370 |
| Lung Adenocarcinoma (TCGA-LUAD) | Bronchus and lung | Acinar Cell Neoplasms, Adenomas and Adenocarcinomas Cystic, Mucinous and Serous Neoplasms | 473 |
| Lower Grade Glioma (TCGA-LGG)^2^ | Brain | Gliomas | 516 |
| Glioblastoma Multiforme (TCGA-GBM)^2^ | Brain | Gliomas (Some samples were marked as not reported) | 140 |
| Adrenocortical carcinoma (TCGA-ACC) | Adrenal gland | Adenomas and Adenocarcinomas | 80 |
| Bladder Urothelial Carcinoma (TCGA-BLCA) | Bladder | Adenomas and Adenocarcinomas, Epithelial Neoplasms, NOS, Squamous Cell Neoplasms, Transitional Cell Papillomas and Carcinomas | 418 |
| Breast Carcinoma (TCGA-BRCA) | Breast | Adenomas and Adenocarcinomas, Adnexal and Skin Appendage Neoplasms, Basal Cell Neoplasms, Complex Epithelial Neoplasms Cystic, Mucinous and Serous Neoplasms, Ductal and Lobular Neoplasms, Epithelial Neoplasms, NOS, Fibroepithelial Neoplasms, Squamous Cell Neoplasms | 791 |
| Cervical Squamous Cell Carcinoma (TCGA-CESC) | Cervical Squamous Cell Carcinoma and Endocervical Adenocarcinoma | Adenomas and Adenocarcinomas, Complex Epithelial Neoplasms Cystic, Mucinous and Serous Neoplasms, Squamous Cell Neoplasms | 307 |
| Colon Adenocarcinoma (TCGA-COAD) | Colon | Adenomas and Adenocarcinomas, Complex Epithelial Neoplasms Cystic, Mucinous and Serous Neoplasms, Epithelial Neoplasms, NOS | 308 |
| Acute Myeloid Leukemia (TCGA-LAML) | Hematopoietic and reticuloendothelial systems | Myeloid Leukemias | 140 |
| Liver Hepatocellular Carcinoma (TCGA-LIHC) | Liver and intrahepatic bile ducts | Adenomas and Adenocarcinomas | 377 |
| Mesothelioma (TCGA- MESO) | Heart, mediastinum, and pleura | Mesothelial Neoplasms | 86 |
| Pancreatic Adenocarcinoma (TCGA-PAAD) | Pancreas | Adenomas and Adenocarcinomas Cystic, Mucinous and Serous Neoplasms, Ductal and Lobular Neoplasms, Epithelial Neoplasms, NOS | 184 |
| Pheochromocytoma and Paraganglioma (TCGA-PCPG) | Adrenal gland | Paragangliomas and Glomus Tumors | 148 |
| Prostate Adenocarcinoma (TCGA-PRAD) | Prostate gland | Adenomas and Adenocarcinomas Cystic, Mucinous and Serous Neoplasms, Ductal and Lobular Neoplasms | 502 |
| Sarcoma (TCGA-SARC) | Connective, subcutaneous and other soft tissues | Fibromatous Neoplasms, Lipomatous Neoplasms, Myomatous Neoplasms, Nerve Sheath Tumors, Soft Tissue Tumors and Sarcomas, NOS, Synovial-Like Neoplasms | 117 |
| Skin Cutaneous Melanoma (TCGA-SKCM) | Skin | Nevi and Melanomas | 104 |
| Stomach Adenocarcinoma (TCGA-STAD) | Stomach | Adenomas and Adenocarcinomas Cystic, Mucinous and Serous Neoplasms | 393 |
| Testicular Germ Cell Tumors (TCGA-TGCT) | Testis | Germ Cell Neoplasms | 150 |
| Thyroid Carcinoma (TCGA-THCA) | Thyroid gland | Adenomas and Adenocarcinomas, Epithelial Neoplasms, NOS | 507 |
| Uterine Corpus Endometrial Carcinoma (TCGA-UCEC) | Corpus uteri | Adenomas and Adenocarcinomas Cystic, Mucinous and Serous Neoplasms, Epithelial Neoplasms, NOS | 438 |
| Uveal Melanoma (TCGA-UVM) | Eye and adnexa | Nevi and Melanomas | 80 |

1 TCGA-KIRC, TCGA-KIRP and TCGA-KICH were grouped as kidney cancer cohort (TCGA-KIDNEY).

2 TCGA-LGG and TCGA-GBM were grouped as glioma cohort (TCGA-GLIOMA).

**Table S2 DNA methylation datasets used for classifier development**

| Abbreviation | Dataset | Sample number in the training set (30%) | Sample number in the validation set (70%) |
| --- | --- | --- | --- |
| OVARY | GEO-GSE102119 | 27 | 64 |
| KIDNEY | Kidney Renal Clear Cell Carcinoma(TCGA-KIRC) | 108 | 216 |
|  | Kidney Renal Papillary Cell Carcinoma(TCGA-KIRP) | 75 | 200 |
|  | Chromophobe Renal Cell Carcinoma (TCGA-KICH) | 16 | 50 |
| LUSC | Lung Squamous Cell Carcinoma (TCGA-LUSC) | 111 | 259 |
| LUAD | Lung Adenocarcinoma (TCGA-LUAD) | 143 | 330 |
| GLIOMA | Lower Grade Glioma (TCGA-LGG) | 163 | 353 |
|  | Glioblastoma Multiforme (TCGA-GBM) | 33 | 107 |
| ACC | Adrenocortical carcinoma (TCGA-ACC) | 25 | 55 |
| BLCA | Bladder Urothelial Carcinoma (TCGA-BLCA) | 126 | 292 |
| BRCA | Breast Invasive Carcinoma (TCGA-BRCA) | 237 | 554 |
| CESC | Cervical Squamous Cell Carcinoma (TCGA-CESC) | 91 | 216 |
| CRC | Colon Adenocarcinoma (TCGA-COAD) | 92 | 216 |
|  | Rectum Adenocarcinoma (TCGA-READ) | 31 | 67 |
| HLM | Acute Myeloid Leukemia (TCGA-LAML) | 41 | 99 |
|  | Diffuse Large B-cell Lymphoma (TCGA-DLBC) | 13 | 35 |
| LIHC | Liver Hepatocellular Carcinoma (TCGA-LIHC) | 113 | 264 |
| MESO | Mesothelioma (TCGA- MESO) | 25 | 61 |
| PAAD | Pancreatic Adenocarcinoma (TCGA-PAAD) | 55 | 129 |
| PCPG | Pheochromocytoma and Paraganglioma (TCGA-PCPG) | 45 | 103 |
| PRAD | Prostate Adenocarcinoma (TCGA-PRAD) | 150 | 352 |
| SARC | Sarcoma (TCGA-SARC) | 35 | 82 |
| SKCM | Skin Cutaneous Melanoma (TCGA-SKCM) | 33 | 71 |
| Upper GI | Stomach Adenocarcinoma (TCGA-STAD) | 118 | 275 |
|  | Esophageal adenocarcinoma (TCGA-EAC) | 26 | 63 |
| TGCT | Testicular Germ Cell Tumors (TCGA-TGCT) | 45 | 105 |
| THCA | Thyroid Carcinoma (TCGA-THCA) | 151 | 356 |
| UC | Uterine Corpus Endometrial Carcinoma (TCGA-UCEC) | 134 | 304 |
|  | Uterine Carcinosarcoma (TCGA-UCS) | 28 | 65 |
| UVM | Uveal Melanoma (TCGA-UVM) | 23 | 57 |
| THYM | Thymoma (TCGA-THYM) | 37 | 87 |
| HN/ESCC | Esophageal Squamous Cell Carcinoma (TCGA-ESCC) | 30 | 66 |
|  | Head and Neck Squamous Cell Carcinoma (TCGA-HNSC) | 158 | 370 |

**Table S3 Infinium 450K methylation array datasets used for classifier validation**

| **Classification** | **Project** | **Pathological classification** | **Sample number** |
| --- | --- | --- | --- |
| PRAD | ICGC-PRAD-CA | Prostate adenocarcinoma | 286 |
| OVARY | ICGC-OV-AU | Ovarian cancer | 83 |
| PAAD | ICGC- PACA-AU | Pancreas ductal adenocarcinoma | 261 |
| LIHC | GEO-GSE56588 | Hepatocellular carcinoma | 224 |
| KIDNEY | GEO-GSE61441 | Clear cell renal cell carcinoma | 46 |
| BRCA | GEO-GSE106360 | Breast cancer | 28 |
| LUSC | GEO-GSE108123 | Lung squamous cell carcinoma | 54 |
| GLIOMA | GEO-GSE50774 | Glioma | 45 |
| CRC | GEO-GSE77954 | Colorectal carcinoma | 25 |

**Table S4 Infinium 850K methylation array datasets used for classifier validation**

| **Classification** | **Project** | **Pathological classification** | **Sample number** |
| --- | --- | --- | --- |
| LUAD | CPTAC - Lung Adenocarcinoma | Lung adenocarcinoma | 229 |
| LUSC | CPTAC - Lung Squamous Cell Carcinoma | Lung squamous cell carcinoma | 108 |
| GLIOMA | CPTAC - Glioblastoma | Glioblastoma | 104 |
| KIDNEY | CPTAC - Renal cell carcinoma | Clear cell renal cell carcinoma | 352 |
| PAAD | CPTAC - Pancreatic duct carcinoma | Pancreas ductal adenocarcinoma, NOS | 148 |
| UC | CPTAC - Endometrioid adenocarcinoma | Uterine corpus endometrial carcinomas | 241 |
| HN/ESCC | CPTAC - HNSC | Head and neck squamous cell carcinomas | 110 |
| CRC | GEO - GSE148766 | Colorectal carcinoma | 36 |
| LIHC | GEO - GSE136380 | Hepatocellular carcinoma | 23 |
| SKCM | GEO - GSE144487 | Skin melanoma | 196 |
| THCA | GEO - GSE121377 | Thyroid cancer | 27 |
| OVARY | GEO-GSE133556, GEO-GSE192967 | High-grade serous ovarian cancer, Non-epithelial ovarian cancer | 131 |
| MESO | GEO-GSE164269 | Mesothelioma | 79 |
| TGCT | GEO-GSE156512 | Testicular germ cell tumor | 21 |
| CESC | <https://gdc.cancer.gov/about-data/publications/CGCI-HTMCP-CC-2020> | Cervical cancer | 120 |

**Figure S1 Mean methylation (β value) of cg16104915 (*HOXA9*) across 23 cancer types**


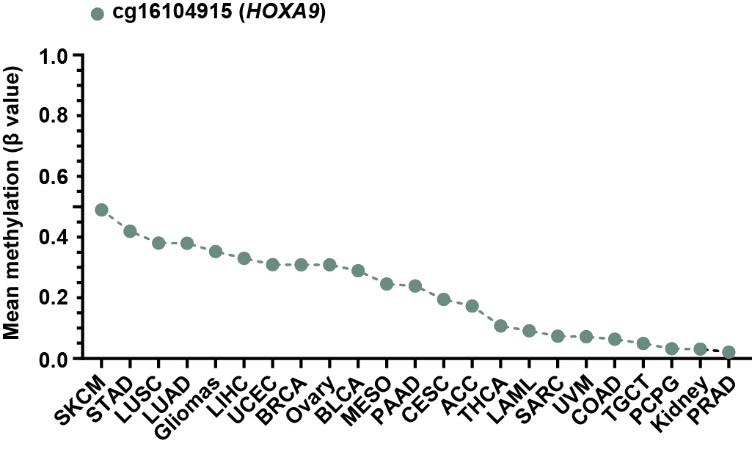


**Figure S2 T-distributed stochastic neighbor embedding (t-SNE) using the methylation profiles of the 200 CpGs across the expanded TCGA/GSE validation cohort (n = 5,923)**


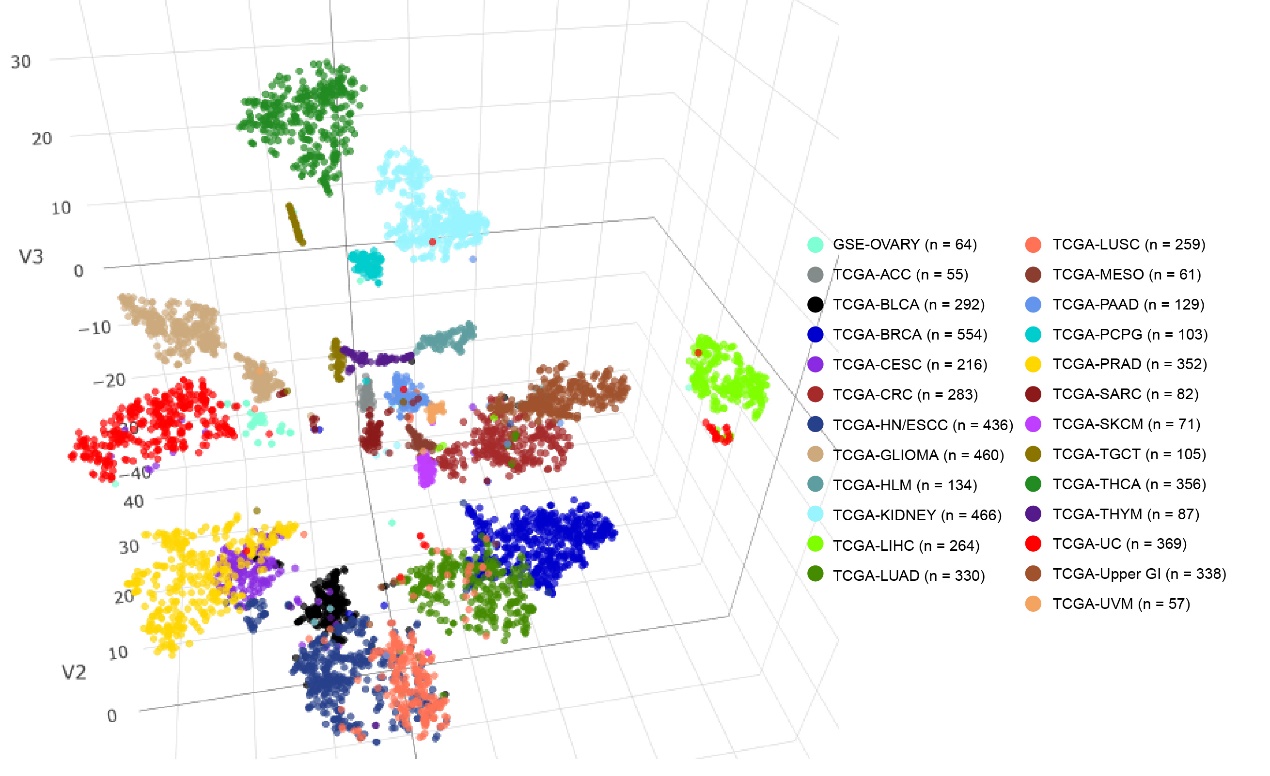

Supplement: Supplementary file 1 — Additional file 1. Supplementary Table 1-4; Supplementary Figure 1-2. [file 13148_2024_1638_MOESM1_ESM.docx]
